# The E3 ubiquitin ligase TRIM39 modulates renal fibrosis induced by unilateral ureteral obstruction through regulating proteasomal degradation of PRDX3

**DOI:** 10.1038/s41420-023-01785-4

**Published:** 2024-01-09

**Authors:** Jun Jian, Yunxun Liu, Qingyuan Zheng, Jingsong Wang, Zhengyu Jiang, Xiuheng Liu, Zhiyuan Chen, Shanshan Wan, Hao Liu, Lei Wang

**Affiliations:** 1 Department of Urology, Renmin Hospital of Wuhan University, Wuhan, 430060 Hubei China; 2 Department of Ophthalmology, Renmin Hospital of Wuhan University, Wuhan, 430060 Hubei China; 3 Department of Urology, The first affiliated hospital of Zhengzhou university, Zhengzhou, 450052 Henan China

**Keywords:** End-stage renal disease, Mechanisms of disease, Renal fibrosis

## Abstract

Renal fibrosis is considered to be the ultimate pathway for various chronic kidney disease, with a complex etiology and great therapeutic challenges. Tripartite motif-containing (TRIM) family proteins have been shown to be involved in fibrotic diseases, but whether TRIM39 plays a role in renal fibrosis remain unexplored. In this study, we investigated the role of TRIM39 in renal fibrosis and its molecular mechanism. TRIM39 expression was analyzed in patients’ specimens, HK-2 cells and unilateral ureteral obstruction (UUO) mice were used for functional and mechanistic studies. We found an upregulated expression of TRIM39 in renal fibrosis human specimens and models. In addition, TRIM39 knockdown was found efficient for alleviating renal fibrosis in both UUO mice and HK-2 cells. Mechanistically, we demonstrated that TRIM39 interacted with PRDX3 directly and induced ubiquitination degradation of PRDX3 at K73 and K149 through the K48 chain, which resulted in ROS accumulation and increased inflammatory cytokine generation, and further aggravated renal fibrosis. It provided an emerging potential target for the therapies of renal fibrosis.

## Introduction

Chronic kidney disease (CKD), characterized by high morbidity and mortality, is a worldwide health problem that can affect billions of individuals [1]. Renal fibrosis is considered the final common irreversible pathway leading to end stage of renal disease (ESRD) in CKD. As a consequence, prevention of renal fibrosis is necessary for inhibiting the progression of CKD. Currently, as the specific mechanism has not been elucidated, there is still no effective intervention to alleviate renal fibrosis.

Oxidative stress has been associated with the progression of CKD [2]. It has been suggested that suppression of oxidative stress would alleviate renal interstitial fibrosis significantly [3]. Normally, oxidative stress occurs when the production of reactive oxygen species (ROS) such as hydroxyl radical (OH), superoxide anion (O^2-^) and hydrogen peroxide (H_2_O_2_) exceeds the capacity of ROS elimination. Unilateral ureteral obstruction (UUO) is a well-established experimental model that imitates the final common tubulointerstitial fibrosis of CKD [4]. TGF-β1 is a pivotal fibrogenic cytokine that mediates fibrosis in UUO. Previous studies have shown that TGF-β1-induced fibroblast activation and differentiation into profibrotic myofibroblast phenotype and matrix production were mediated by the ROS-MAPK signaling pathway [5]. In response to ROS, renal tubulointerstitial fibroblasts were converted into myofibroblasts, in addition, renal tubular epithelial-mesenchymal transformation (EMT) was activated, and ultimately led to renal fibrosis [6]. More and more studies have found that mitochondrial damage was closely related to renal fibrosis. Mitochondrial destruction and reduced mitochondrial DNA were found in TGF-induced renal fibrosis, and mitochondrial damage was also reversed after renal fibrosis was reduced [7]. However, the specific mechanism between mitochondrial damage and renal fibrosis remains unclear. It was found that inhibiting the generation of ROS significantly increased the mitochondrial membrane potential (MMP) and reduced mitochondrial damage, and ameliorated renal fibrosis [8]. Mitochondria is the most important energy storage and supply site in cells, however, aging and defective mitochondria can produce toxic ROS. Besides, oxidative stress is an important factor inducing mitochondrial dysfunction, ROS accumulation, the changes in mitochondrial membrane permeability, leading to a loss of MMP and inducing cell necrosis and apoptosis [9]. In summary, ROS is the main cause of mitochondria dysfunction in UUO model, and promotes the development of renal fibrosis. Reducing ROS production may be an important treatment of renal fibrosis.

The peroxiredoxin (PRDX) family has peroxidase activity to destroy peroxides. There are six different members in PRDX family. Peroxiredoxin3 (PRDX3), located in the mitochondria, plays a major role in antioxidant stress by catalyzing reduction of hydrogen peroxide (H_2_O_2_) [10]. It inactivates H_2_O_2_ by binding to the conserved cysteine peroxide in the amino terminal region of the protein. Previous studies have reported that transgenic mice over-expressing PRDX3 reduced H_2_O_2_ production in mitochondria, while PRDX3 knockout mice showed more ROS accumulation and oxidative damage [11, 12]. Moreover, PRDX3 deacetylation could reduce mitochondrial oxidative stress injury and apoptosis caused by ischemia-reperfusion injury [13]. Since the increase of ROS was a key pathological process of UUO, in this study, we investigated that whether PRDX3 regulated renal fibrosis by affecting oxidative stress.

Widely present in eukaryotic cells, ubiquitination exists as an important post-translational modification and plays a vital role in various life processes. As an E3 ubiquitin ligase, TRIM39 consist of a ring domain, one or two B-boxes and a coiled-coil region [14]. The ring domain has E3 ubiquitin activity, which is essential for TRIM39 to perform various biological functions such as regulation of growth and development, cell proliferation, apoptosis, immunity, inflammation, antiviral and tumorigenesis [15–18]. In recent years, the role of TRIM protein family in the progression of fibrosis in various organs has been discovered. Overexpression of TRIM6 increased ubiquitination levels of TSC1 and TSC2 and led to activation of the mTORC1 pathway, thus aggravating renal fibrosis [19]. In addition, TRIM18 promoted kidney inflammation and fibrosis through the PTP1B/STAT3 pathway, aggravating diabetic kidney disease [20]. However, the role of TRIM39 in renal fibrosis has not been studied. In this study, we found that TRIM39 expression was elevated in UUO, and its knockout alleviated UUO-mediated renal fibrosis. Furthermore, we also demonstrated that TRIM39 reduced H_2_O_2_ clearance through ubiquitination degradation of PRDX3, leading to ROS accumulation and aggravating renal fibrosis.

## Results

### TRIM39 expression was elevated in response to UUO and TGF-β1

**Fig. 1:**
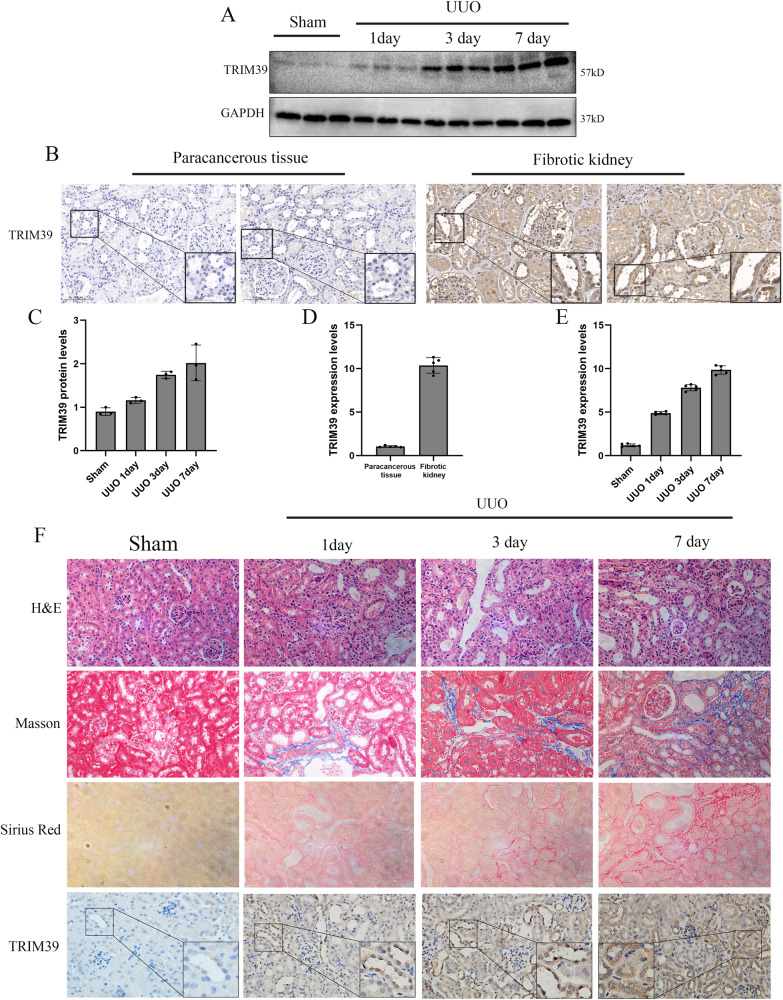
TRIM39 is highly expressed in renal fibrosis and positively correlated with the severity of renal fibrosis. **A** Representative Western blot images of TRIM39 levels in Sham and UUO mice. *n* = 6. **B, D** Representative photographs and quantification of immunohistochemical staining of TRIM39 in paracancerous tissues and fibrotic human kidneys specimens. **C** Bar graph showing the fold changes of TRIM39 relative to sham group from three independent samples. **E** Quantification of immunohistochemical staining of TRIM39 in sham or UUO group. **F** Representative images of hematoxylin and eosin (H&E), Masson, Sirius Red and immunohistochemical staining of TRIM39 in sham or UUO renal. *n* = 6. The degree of fibrosis was assessed and quantified in five randomly selected microscopic fields. Each experiment was repeated at least three times independently.

TRIM39 expression was detected in vivo and in vitro models. As shown, it was significantly increased in UUO model and with the extension of obstructed period, it continued to elevate (Fig. 1A, C). Fibrotic kidney tissues and healthy renal tissues were collected from patients underwent surgical treatment. The results showed that TRIM39 expression was significantly increased in fibrotic kidney (Fig. 1B, D). In in vitro model, compared with control group, TRIM39 protein level was significantly higher in TGF-β1 group, especially as the concentration of TGF-β1 increased (Fig. 3A, B). We further explored the potential mechanism of TGF-β1 up-regulated TRIM39 expression, it was found that p-Smad2, p-Smad3 and p-Smad4 expression were significantly increased while p-Smad7 was decreased in TGF-β1 group (Supplementary Fig. S3A, B). Knockdown of Smad3 rather than Smad2, Smad4 or Smad7 reduced TRIM39 expression induced by TGF-β1 markedly (Supplementary Fig. S3C–R), which indicated that TGF-β1 might up-regulate TRIM39 expression by activating Smad3.

In H&E, Masson, and Sirius Red staining, the UUO group showed more renal fibrosis than the sham group. The immunohistochemical staining indicated that TRIM39 expression in UUO group was increased obviously, and positively related with the extension of obstructed period (Fig. 1E, F). Furthermore, fluorescence staining showed that TRIM39 expression in TGF-β1 group was remarkably increased in a concentration-dependent manner (Fig. 3G, I). Therefore, these results suggested that TRIM39 might be involved in the development of renal fibrosis after UUO.

### TRIM39 knockout alleviated renal fibrosis induced by UUO

**Fig. 2:**
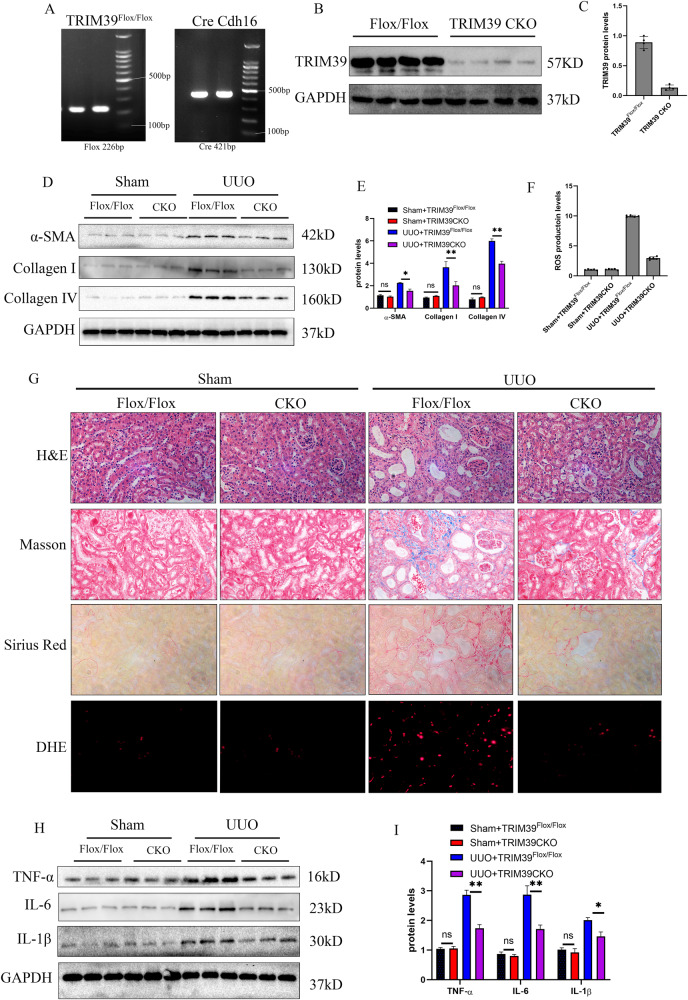
TRIM39 knockout alleviated renal fibrosis induced by UUO. **A**The PCR of gene expression of TRIM39^Flox/Flox^ and Cre Cdh16 mice, gel electrophoresis imaging showed that the length of Cre gene amplification product was 421 bp, and the length of Flox gene amplification product was 226 bp. **B, C** Representative western blot and quantification images of TRIM39 expression in TRIM39^Flox/Flox^ and TRIM39 conditional knockout (CKO) mice. **D, E, H, I** Representative western blot and quantification images of α-SMA, collagen I and IV, TNF-α, IL-6 and IL-1β expression in kidneys of the four groups. **F** Bar graph showing the fold changes of ROS production relative to sham group from three independent samples. **G** Representative images of H&E, Masson, Sirius Red and DHE staining of kidney tissues of the four groups. The degree of fibrosis was assessed and quantified in five randomly selected microscopic fields. All data were showed as mean ± SEM, Two-way analysis of variance was used. *n* = 6, *P < 0.05, **P < 0.01, ***P < 0.001. Each experiment was repeated at least three times independently.

To investigate the effect of TRIM39 on renal fibrosis, CKO mice were obtained using TRIM39^Flox/Flox^ and Cre Cdh16 mice. The gel electrophoresis imaging showed that the length of Cre gene amplification product was 421 bp, and the length of Flox gene amplification product was 226 bp (Fig. 2A). Western blot showed that TRIM39 protein level was significantly reduced in CKO mice (Fig. 2B, C). In H&E, Masson and Sirius Red staining, the kidney damage and collagen deposition were dramatically decreased in CKO mice compared with TRIM39^Flox/Flox^ mice subjected to UUO (Fig. 2G). DHE staining showed that the ROS content was significantly decreased in CKO mice subjected to UUO (Fig. 2F, G). Besides, we also examined factors related with fibrosis and inflammation, such as α-SMA, collagen I and IV, TNF-α, IL-6 and IL-1β, and the results showed that the above mRNA and protein levels in CKO mice were observably less than TRIM39^Flox/Flox^ mice subjected to UUO (Fig. 2D, E, H, I; Supplementary Fig. S4A). In conclusion, these results demonstrated that TRIM39 might aggravate UUO-induced renal fibrosis by enhancing the production of inflammation factors.

### Knockdown of TRIM39 inhibited fibrosis and mitochondrial ROS in vitro

**Fig. 3:**
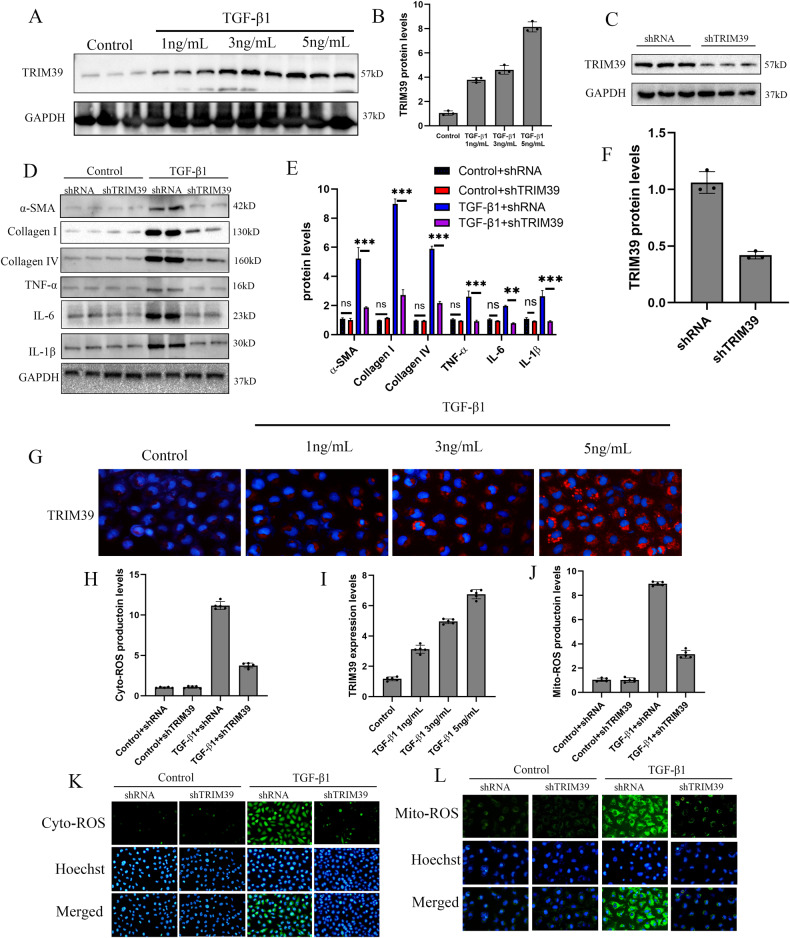
Knockdown of TRIM39 inhibited fibrosis and mitochondrial ROS in vitro. **A, B** Representative western blot and quantification images of TRIM39 expression in HK-2 cells treated with TGF-β1 or in control group. **C, F** Representative western blot images and quantification of TRIM39 expression in HK-2 cells treated with shRNA or shTIRM39. **D, E** Representative western blot and quantification images of α-SMA, collagen I and IV, TNF-α, IL-6 and IL-1β expression in the four HK-2 cells groups. **G, I** Immunofluorescence staining of TRIM39 and its quantification graphs in HK-2 cells treated with TGF-β1 at concentrations of 1,3 and 5 ng/L or in control group. **H, K** Representative images showing intracellular ROS stained with DCFH-DA dye in the indicated groups from three independent experiments. **J, L** Representative images showing mitochondria ROS stained with Mitochondrial ROS production rate test kit in the indicated groups from three independent experiments. The ROS production was assessed and quantified in five randomly selected microscopic fields. All data were showed as mean ± SEM, Two-way analysis of variance was used. *P < 0.05, **P < 0.01, ***P < 0.001. Each experiment was repeated at least three times independently.

To verify the effect of TRIM39, short hairpin RNA (shRNA) targeting TRIM39 were annealed and cloned into PLKO.1 plasmid and transfected HK-2 cells with lentivirus. Western blot was performed to verify the TRIM39 knockdown efficiency in vitro (Fig. 3C, F). The results showed that mRNA and protein levels of α-SMA, collagen I and IV, TNF-α, IL-6 and IL-1β in TRIM39 knockdown group were markedly lower than those in the shRNA group (Fig. 3D, E; Supplementary Fig. S4B, C). Then, the results showed that TGF-β1 stimulation could induce ROS production of mitochondria and TRIM39 knockdown reduced ROS level (Fig. 3H, J–L). Overall, these results suggested that TRIM39 knockdown could alleviate fibrosis by reducing the production of inflammation factors and mitochondrial ROS.

### Over-expression of PRDX3 alleviated renal UUO-mediated renal fibrosis

According to our mass spectrometry results, the thioredoxin-dependent peroxide reductase interacted with TRIM39 (Supplementary Fig. S1A). The peroxiredoxin (PRDX) family, serving as the thioredoxin-dependent peroxide reductase, plays a vital role in eliminating ROS. PRDX3 was the most abundant and efficient ROS elimination enzyme in mitochondria and targeted nearly 90% of the H_2_O_2_ produced in mitochondrial matrix [13]. Coomassie bright blue staining was performed to visualize the protein interacted with TRIM39, and then we produced HA-tagged TRIM39 and Flag-tagged PRDX3 recombinant proteins for Co-IP assays. The results showed that HA-TRIM39 coimmunoprecipitated with Flag-PRDX3, indicating that TRIM39 interacted with PRDX3 in vitro (Supplementary Fig. S1B, C).

**Fig. 4:**
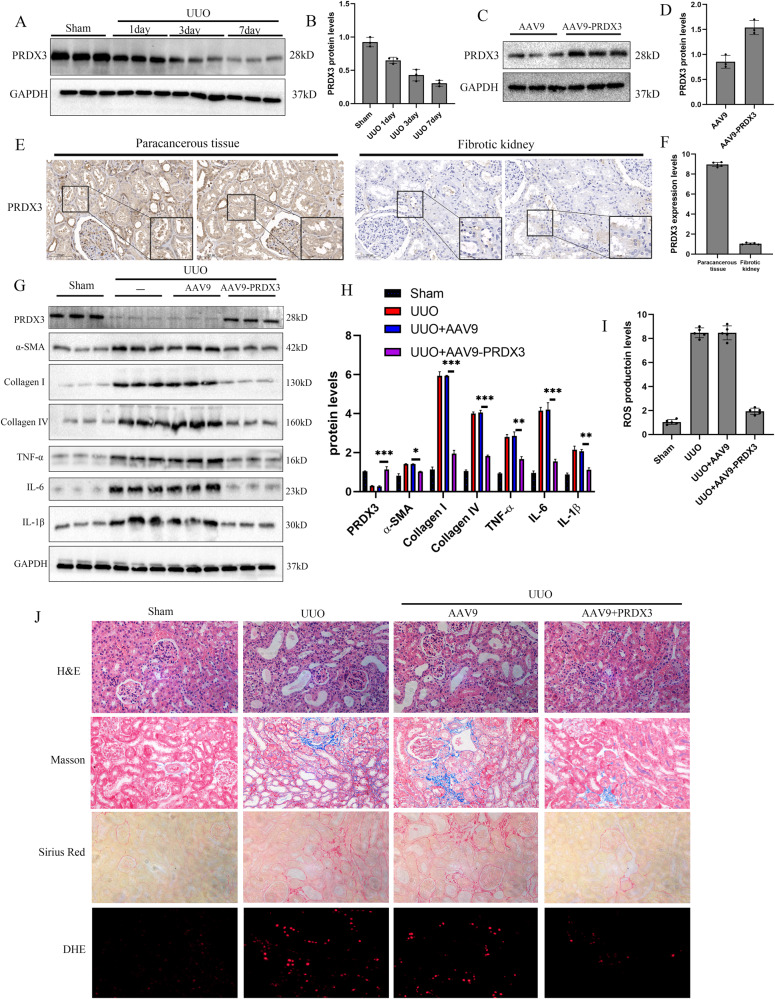
Over-expression of PRDX3 alleviated renal UUO-mediated renal fibrosis. **A, B** Representative western blot and quantification images of PRDX3 levels in Sham and UUO mice. *n* = 6. **C, D** Representative western blot and quantification images of PRDX3 levels in kidney of AAV9 and AAV9-PRDX3 treated mice. *n* = 6. **E, F** Representative photographs of immunohistochemical staining of PRDX3 and its quantification images in paracancerous tissues and fibrotic human kidneys specimens. **G, H** Representative western blot and quantification images of α-SMA, collagen I and IV, TNF-α, IL-6 and IL-1β expression in kidneys of the four mouse groups. GAPDH was used for normalization. *n* = 6. **I** Bar graph showing the fold changes of ROS production relative to sham group from three independent samples. **J** Representative images of H&E, Masson, Sirius Red and DHE staining in kidneys of the four mouse groups. *n* = 6. The degree of fibrosis and oxidative stress was assessed and quantified in five randomly selected microscopic fields. All data were showed as mean ± SEM, Two-way analysis of variance was used. * *P* < 0.05, **P < 0.01, ***P < 0.001. Each experiment was repeated at least three times independently.

Next, we explored the role of PRDX3 in renal fibrosis. Western blot showed that PRDX3 expression was decreased after UUO (Fig. 4A, B). Also, PRDX3 expression was significantly reduced in human fibrotic kidney (Fig. 4E, F). Next, we treated mice with adeno-associated virus 9 (AAV9) carrying PRDX3 (AAV-PRDX3) via tail vein injection, and western blot was used to verify the efficiency (Fig. 4C, D). The results showed that renal fibrosis, collagen deposition and ROS production in PRDX3 over-expressed group were less than control group (Fig. 4I, J). Consistent with it, western blot and real-time quantitative PCR showed that the levels of α-SMA, collagen I and IV, TNF-α, IL-6 and IL-1β in PRDX3 over-expressed group were decreased (Fig. 4G, H; Supplementary Fig. S4D). Taken together, these results manifested that over-expression of PRDX3 could alleviate renal fibrosis by decreasing inflammation related factors in UUO mice.

### PRDX3 over-expression inhibited TGF-β1-stimulated fibrosis in vitro

**Fig. 5:**
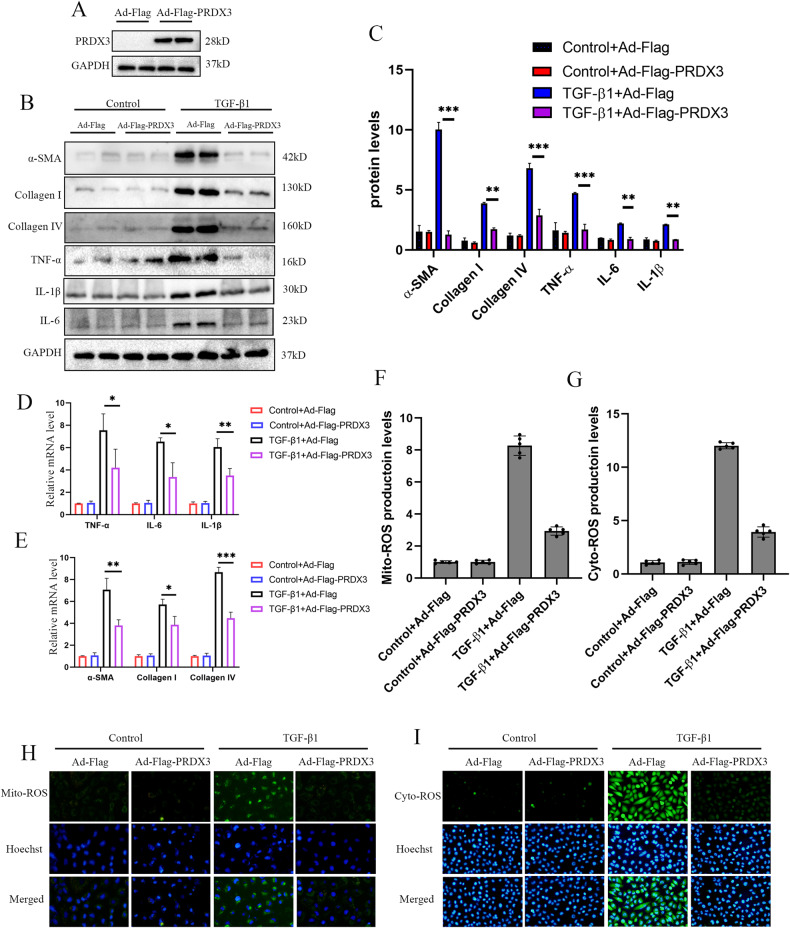
PRDX3 over-expression inhibited TGF-β1-stimulated fibrosis in vitro. **A** Representative western blot images of PRDX3 protein levels in HK-2 cells treated with adenovirus-Flag or adenovirus-Flag-PRDX3. **B**, **C** Representative western blot and quantification images of α-SMA, collagen I and IV, TNF-α, IL-6 and IL-1β expression in HK-2 cells of each group. **D**, **E** Real-time PCR analysis for the mRNA levels of α-SMA, collagen I and IV, TNF-α, IL-6 and IL-1β in HK-2 cells of each group. **F, H** Representative images of the mitochondria ROS production in HK-2 cells of each group. **G, I** Representative images of the intracellular ROS production in HK-2 cells of each group. The ROS production was assessed and quantified in five randomly selected microscopic fields. All data were showed as mean ± SEM, Two-way analysis of variance was used. *P < 0.05, **P < 0.01, ***P < 0.001. Each experiment was repeated at least three times independently.

The regulation of PRDX3 on renal fibrosis was further investigated in HK-2 cells. PRDX3 was over-expressed in HK-2 cells(Fig. 5A). As shown, PRDX3 over-expression prominently blocked α-SMA, collagen I and IV, TNF-α, IL-6 and IL-1β expression in HK-2 cells at both the mRNA and protein levels (Fig. 5B–E). Fluorescence staining indicated that as PRDX3 over-expressed, the ROS production in cytoplasm and mitochondria was weakened (Fig. 5F–I). Therefore, these results declared that over-expression of PRDX3 attenuated fibrosis by restraining the production of mitochondrial ROS and inflammation factors.

### TRIM39 aggravated renal fibrosis dependent on PRDX3 in UUO mice

To further verify the interaction between TRIM39 and PRDX3, immunofluorescence staining was performed, and the results showed that TRIM39 colocalized with PRDX3 (Supplementary Fig. S2A). Next, the glutathione S-transferase (GST) pull-down assay showed that TRIM39 and PRDX3 interacted directly (Supplementary Fig. S2B–C). To explore the specific binding domain of TRIM39 and PRDX3 interaction, several domain deletion mutants of TRIM39 (Δ R, Δ B, Δ SPRY) and PRDX3 (1-100 amino acids (aa), 1-150aa, 101-150aa, 101-256aa) were constructed for Co-IP experiments. And our results suggested that the SPRY domain of TRIM39 reacted with the 101-256aa region of PRDX3 (Supplementary Fig. S2D–E).

**Fig. 6:**
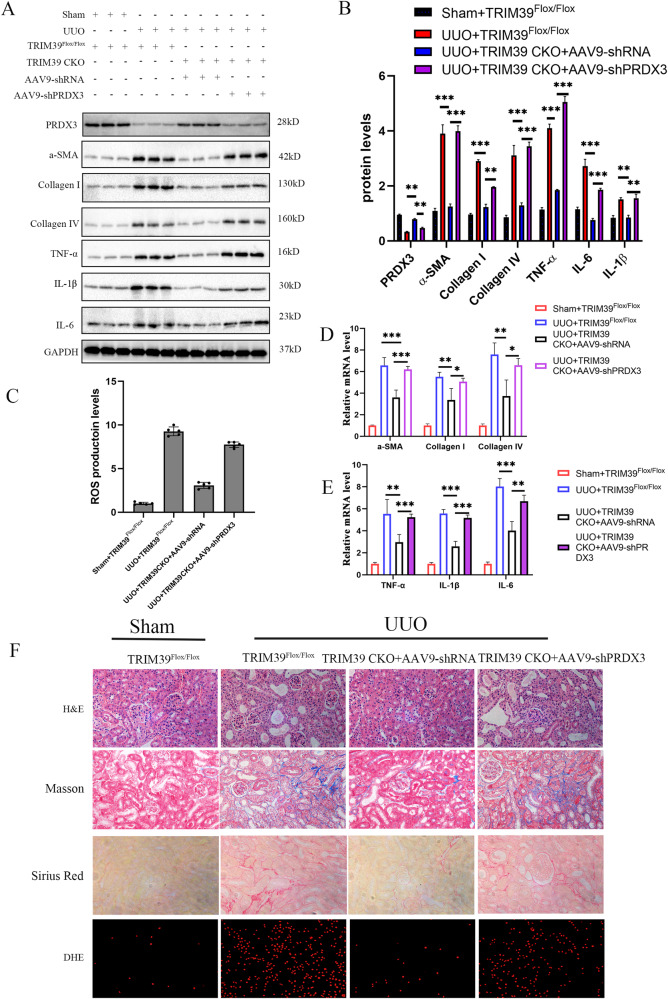
TRIM39 aggravated renal fibrosis dependent on PRDX3 in UUO mice. **A, B** Representative western blot and quantification images of PRDX3, α-SMA, collagen I and IV, TNF-α, IL-6 and IL-1β expression in kidneys of the four mouse groups. **C** Bar graph showing the fold changes of ROS production relative to sham group from three independent samples. **D, E** Representative Real-time PCR analysis images for the relative mRNA levels of α-SMA, collagen I and IV, TNF-α, IL-6 and IL-1β in kidneys of the four mouse groups. **F** Representative images of H&E, Masson, Sirius Red and DHE staining in kidneys of the four mouse groups. The degree of fibrosis and oxidative stress was assessed and quantified in five randomly selected microscopic fields. All data were showed as mean ± SEM, Two-way analysis of variance was used. *n* = 6, *P < 0.05, **P < 0.01, ***P < 0.001. Each experiment was repeated at least three times independently.

To further explore the relationship between TRIM39 and PRDX3 in renal fibrosis, we performed a tail-vein injection of the shPRDX3 in CKO mice and TRIM39^Flox/Flox^ mice. Western blot was performed to examine the PRDX3 expression in each group (Fig. 6A). As shown, PRDX3 low-expression increased α-SMA, collagen I and IV, TNF-α, IL-6 and IL-1β mRNA and protein levels in CKO mice (Fig. 6A, B, D, E). Moreover, in H&E, Masson and Sirius Red staining, the kidney damage and collagen deposition were dramatically increased in PRDX3 low-expression group (Fig. 6F). Also, DHE staining showed that ROS production increased significantly during PRDX3 low-expression(Fig. 6C, F). Therefore, these results demonstrated that TRIM39 aggravated renal fibrosis in UUO mice depending on PRDX3.

### TRIM39 regulated fibrosis and oxidative stress in a PRDX3 manner

**Fig. 7:**
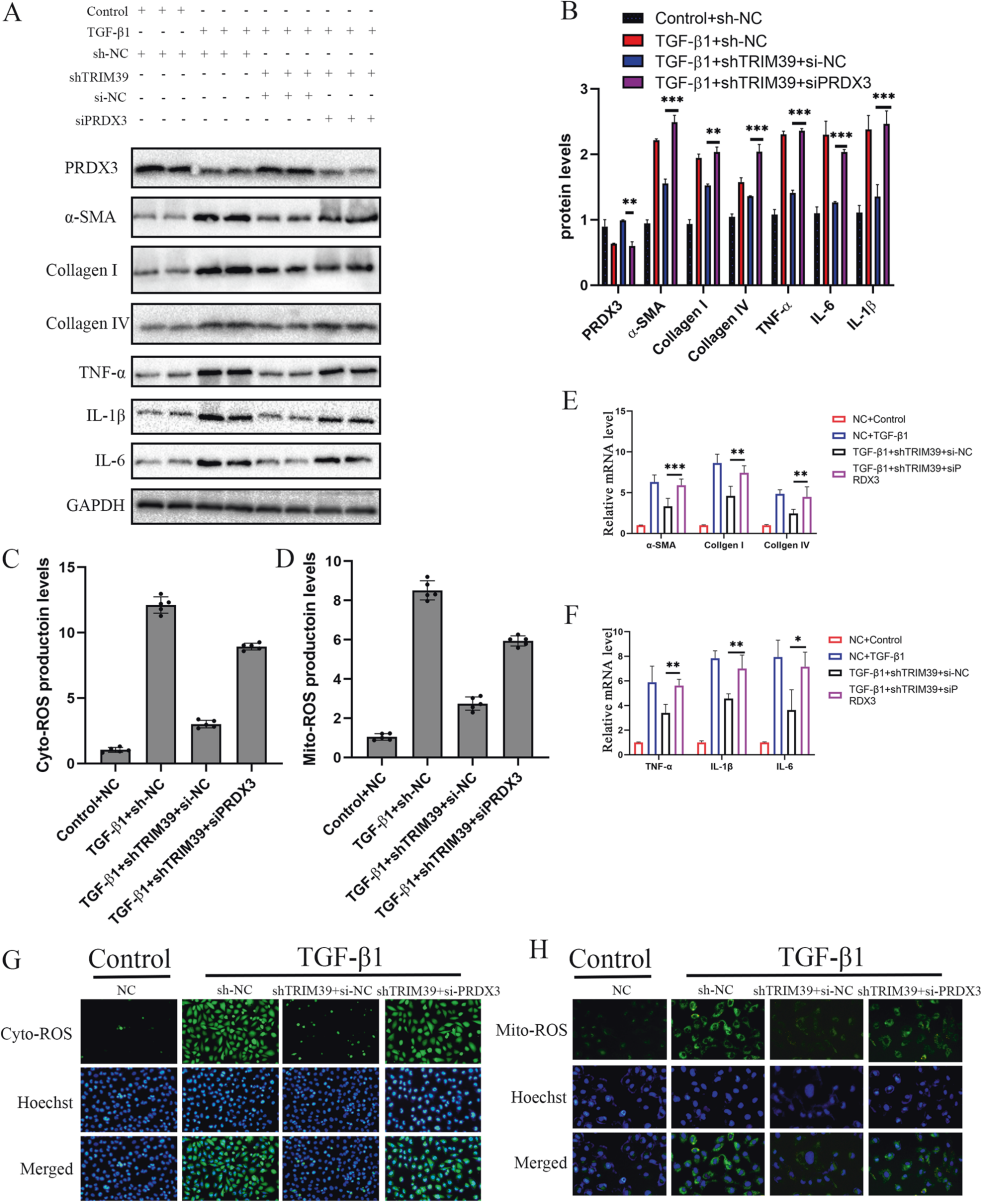
TRIM39 regulated fibrosis and oxidative stress in a PRDX3 manner. **A, B** Representative western blot and quantification images of PRDX3, α-SMA, collagen I and IV, TNF-α, IL-6 and IL-1β expression in HK-2 cells of four groups. **C, D** Representative quantification images of the ROS production in HK-2 cells or in mitochondria. **E, F** Representative Real-time PCR analysis images for the relative mRNA levels of α-SMA, collagen I and IV, TNF-α, IL-6 and IL-1β in HK-2 cells of four groups. **G, H** Representative images of the ROS production in HK-2 cells or in mitochondria. The ROS production was assessed and quantified in five randomly selected microscopic fields. All data were showed as mean ± SEM, Two-way analysis of variance was used. *P < 0.05, **P < 0.01, ***P < 0.001. Each experiment was repeated at least three times independently.

To further investigate whether TRIM39 regulated renal fibrosis and oxidative stress through PRDX3, we knocked down these two genes in HK-2 cells. The results showed that the expression of α-SMA, collagen I and IV, TNF-α, IL-6 and IL-1β were obviously suppressed in the TRIM39 knockdown group. However, this effect was significantly reversed, while PRDX3 further knockdown (Fig. 7A, B, E, F). Knockdown of TRIM39 dramatically reduced the production of ROS induced by TGF-β1, and this effect was reversed after PRDX3 further knockdown (Fig. 7C, G). The ROS production in mitochondria were consistent with the above trend (Fig. 7D, H). In general, these results suggested that TRIM39 regulated fibrosis and oxidative stress in a PRDX3 manner in vitro.

### TRIM39 ubiquitinated PRDX3 and promoted degradation of PRDX3 in vivo and vitro

**Fig. 8:**
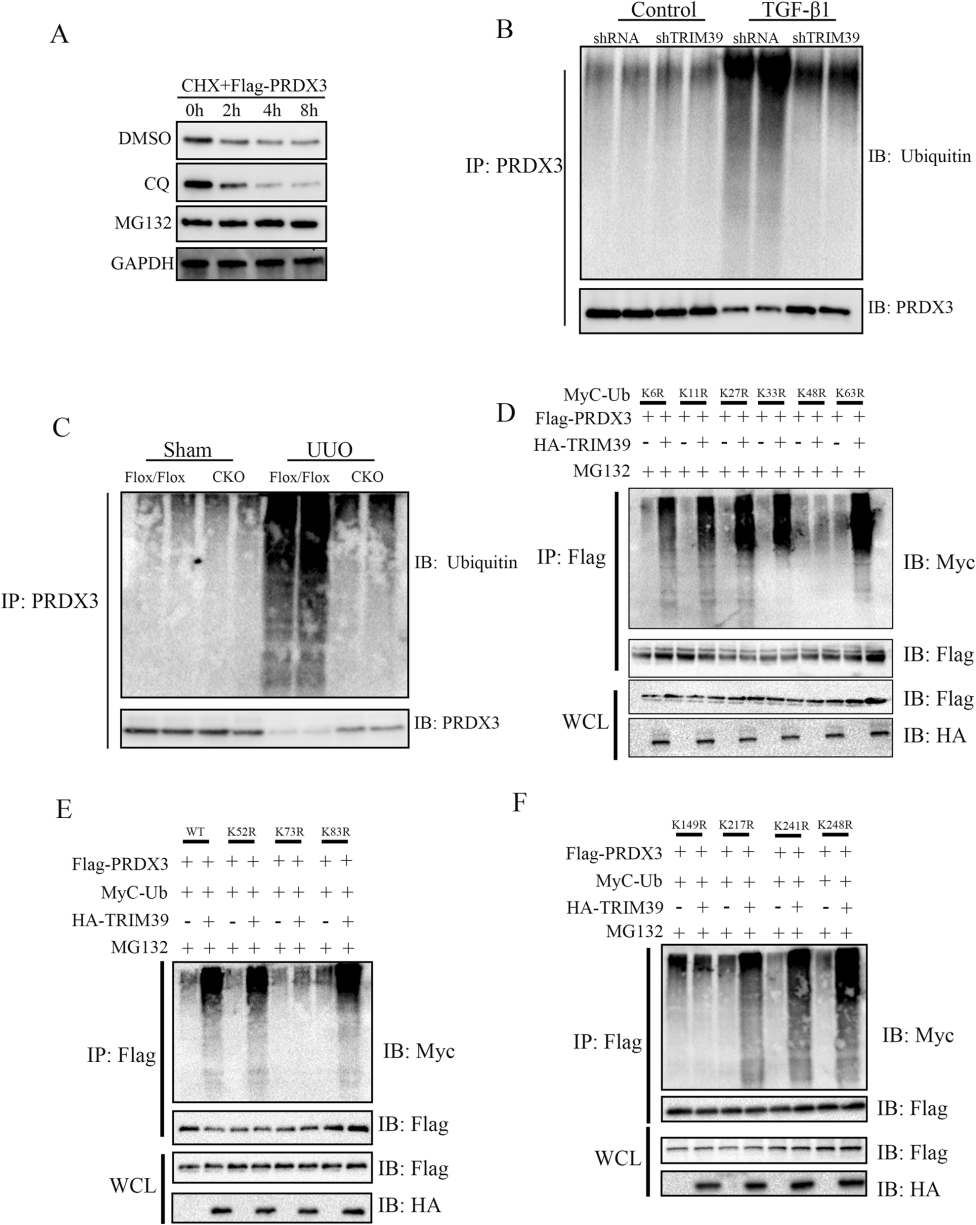
TRIM39 induced PRDX3 ubiquitination degradation at lysine K73 and K149 through K-48 chain. **A** Representative western blot images of PRDX3 degradation in HK-2 cells treated with Cycloheximide (CHX) follow by DMSO, CQ or MG132. **B** Co-IP analysis of PRDX3 ubiquitination in HK-2 cells of TRIM39 knockdown group or control group. **C** Co-IP analysis of PRDX3 ubiquitination in UUO mice of TRIM39 knockout group or control group. **D** Co-IP analysis of PRDX3 ubiquitination in HEK293 cells transfected with plasmids expressing Flag-PRDX3, HA-TRIM39, Myc-ubiquitin, Myc-ubiquitin(K6R), Myc-ubiquitin(K11R), Myc-ubiquitin(K27R), Myc-ubiquitin(K33R) Myc-ubiquitin(K48R), or HA-ubiquitin(K63R). **E, F** Co-IP analysis of the polyubiquitination of PRDX3 and its mutants in HEK293 cells transfected with plasmids encoding Flag-PRDX3 (WT or mutant), plus HA-TRIM39, Myc-ubiquitin and MG132. Each experiment was repeated at least three times independently.

HK-2 cells were treated with Cycloheximide (CHX) and western blot showed that PRDX3 protein degradation in MG132 group was significantly reduced at 2, 4, 8 h, which indicated that the degradation of PRDX3 was related to proteasome (Fig. 8A). Since proteasomal degradation of proteins was mostly related to ubiquitination modification, we speculated that TRIM39 might aggravate renal fibrosis by regulating ubiquitination degradation of PRDX3. To verify this hypothesis, we tested the effects of TRIM39 deletion on PRDX3 ubiquitination in vivo and in vitro. The results showed that PRDX3 ubiquitination was markedly increased in HK-2 cells with TGF-β1 treatment. However, TGF-β1 stimulated ubiquitination of PRDX3 was significantly weakened while TRIM39 knocked down (Fig. 8B). Consistently, TRIM39 knockout alleviated PRDX3 ubiquitination in UUO mice (Fig. 8C). Collectively, these results indicated that TRIM39 destabilized PRDX3 via the ubiquitin-proteasome pathway.

### TRIM39 ubiquitinated PRDX3 at lysine K73 and K149

To investigate the type of TRIM39-mediated PRDX3 polyubiquitin, we transfected HEK293 cells with plasmids expressing MyC-labeled mutant ubiquitin, in which one of the lysine (K) residues of ubiquitin was replaced by arginine (R) to construct the K6R, K11R, K27R, K33R, K48R and K63R mutant. And it showed that PRDX3 ubiquitination was obviously decreased in K48R group, which suggested that TRIM39 induced PRDX3 ubiquitination degradation through the K48-linked chain (Fig. 8D). Next, we explored the lysine residues associated with TRIM39-mediated ubiquitination of PRDX3 protein. We replaced all lysine residues with arginine to construct single lysine mutant of PRDX3, such as K52R, K73R, K83R, K149R, K217R, K241R and K248R. Co-IP assay showed that when K73 and K149 were mutated into R, the ubiquitination of PRDX3 was markedly reduced, suggesting that TRIM39 mediated PRDX3 ubiquitination at K73 and K149 (Fig. 8E-F). In conclusion, these results revealed that TRIM39 induced PRDX3 degradation at lysine K73 and K149 through K48-linked ubiquitin to aggravate renal fibrosis.

## Discussion

Renal fibrosis causes a progressive and irreversible impairment of renal function and is the ultimate consequence of all chronic kidney disease, eventually leading to end-stage renal disease. Due to its high morbidity and still no effective treatment, renal fibrosis places a substantial burden on health care worldwide [21]. Therefore, the exploration of new therapeutic strategies is urgent and essential. Our study was the first to demonstrate the expression of TRIM39 was increased in renal fibrosis, while PRDX3 was decreased. Meanwhile, TRIM39 participated in the regulation of renal fibrosis through interacting with PRDX3 and mediating its ubiquitination. Further, we found that TRIM39 mediated the ubiquitination degradation of PRDX3 at K73 and K149 through K48 chain.

As one of the largest families of E3 ubiquitin ligases, the TRIM family proteins have been investigated for its multiple functions in cellular processes, including inflammation, carcinogenesis, apoptosis and metabolism [22]. Prior reports have shown that TRIM family proteins play an important role in kidney fibrotic disease, TRIM72 [23], TRIM6 [19] and TRIM18 [20] were involved in the development of renal fibrosis by regulating downstream inflammatory pathways. However, the role of TRIM39 in renal fibrosis and its underlying mechanisms still remain unclear. The current study revealed a previously unrecognized biological function of TRIM39 in aggravating renal fibrosis. We found that TRIM39 expression was significantly increased in fibrotic kidney tissue compared with paracancerous normal tissue, which suggested that TRIM39 might be involved in the formation of renal fibrosis. Next, we generated UUO mice and TGF-β1 treated HK-2 cells to establish renal fibrosis models in vivo and in vitro. Consistent with human kidney specimens, TRIM39 expression was markedly increased in renal fibrosis models. These results indicated that TRIM39 might play a crucial role in the progression of renal fibrosis. Since TGF-β1 mediated the activation of phosphorylation of Smad family, especially Smad3, was a classic pathway for its induction of renal fibrosis [24], we speculated that this may be related to its up-regulation of TRIM39 expression. Consistent with previous studies that found that Smad2,Smad3,Smad4 and Smad7 might be involved in TGF-β1-mediated renal fibrosis [24–28], our study observed alterations in the phosphorylation activation of Smad2,Smad3,Smad4 and Smad7 in TGF-β1 treated HK-2 cells. To explore the potential mechanism of TGF-β1 up-regulation of TRIM39 expression, we performed knockdown on these Smad family proteins, and the results showed that Smad3 knockdown significantly reversed TGF-β1-induced TRIM39 expression. It indicated that TGF-β1 might up-regulate TRIM39 expression by activating Smad3. Interestingly, the phosphorylation activation of Smad2 and Smad4 in TGF-β1-induced renal fibrosis was similar to that of Smad3, but their knockdown has no significant effect on TRIM39 expression. We speculated that it might be related to the fact that Smad 2 and Smad4 does not have DNA-binding domains, but acts as a regulator of Smad3-based gene transcription [29].Of course, due to the complexity of the mechanism of TGF-β1-induced renal fibrosis, our findings cannot yet determine whether TGF-β1-induced up-regulation of TRIM39 expression are related to non-Smad pathways, which may be further studied in the future.

UUO mice had abundant deposition of collagen fibers and increased fibrosis-related products and inflammatory factors such as α-SMA, collagen I and IV, TNF-α, IL-6 and IL-1β, which were obviously decreased by TRIM39 knockout. We also found that ROS production was notably increased in UUO mice, and it was reversed by TRIM39 knockout. Previous studies indicated that UUO activated the renin-angiotensin system to further activate NOXs and produce ROS under the mechanical pressure [30]. ROS have been reported to be involved in fibroblast activation, epithelial cell migration, and proliferation of mesangial cells [31]. The NOX4-derived ROS was involved in the inflammation and the subsequent renal fibrosis process of diabetic nephropathy [32]. And it was found that the inhibition of ROS production could significantly reduce renal fibrosis [33], which inspired us to further investigate the targets and molecular mechanisms of TRIM39 regulating oxidative stress.

To explore the TRIM39 function during renal fibrosis development, we focused on oxidative stress as it played essential role in renal fibrosis. We identified downstream protein that interacted with TRIM39 by mass spectrometry, among which the thioredoxin-dependent peroxide reductase was related to the oxidative stress pathway. As the TGF-β1 treated HK-2 cells showed obviously ROS generation in mitochondria and PRDX3 was the most abundant and efficient ROS elimination enzyme in mitochondria [13], we investigated whether TRIM39 affects oxidative stress by interacting with PRDX3. The Co-IP assays results showed that TRIM39 coimmunoprecipitated with PRDX3 which indicated that TRIM39 interacted with PRDX3.

To further determine whether PRDX3 played a role in renal fibrosis, we observed the PRDX3 expression in human specimen. It was interesting to note that PRDX3 expression was obviously decreased in fibrotic kidney compared to paracancerous tissue. And the same results were obtained from renal fibrosis models in vitro and in vivo. Subsequently, we over-expressed PRDX3 in vivo and in vitro models and it was found that ROS production, fibrosis related factors and inflammatory cytokine induced by UUO and TGF-β1 were dramatically reduced. To investigate the mechanism of these effects, we injected AAV9-shPRDX3 to tail vein of TRIM39 CKO mice. Interestingly, the protective effect of TRIM39 knockout on UUO was reversed by down-regulation of PRDX3 expression, which indicated that TRIM39 might regulate renal fibrosis and oxidative stress in a PRDX3 manner. Our results showed that mice with PRDX3 knockdown were more susceptible to UUO-induced renal fibrosis, but whether the same phenomenon occurs in mice with deletion of PRDX3 gene remains unclear. Previous study found that PRDX3 knockout aggravated myocardial fibrosis and ventricular remodeling after myocardial infarction in mice [34]. Based on this result, it was reasonable to believe that mice with PRDX3 knocked out may be more vulnerable to renal fibrosis when subjected to UUO. We then explored whether other proteins mediated the TRIM39 interacted with PRDX3. The immunofluorescence staining showed that TRIM39 colocalized with PRDX3, and the GST pull down assay reveled that TRIM39 and PRDX3 interacted directly. We also demonstrated the binding domains responsible for the TRIM39-PRDX3 interaction by Co-IP assay.

To further investigate the molecular mechanism of TRIM39 regulating PRDX3, we explored the degradation manner of PRDX3, and it revealed that PRDX3 degradation related to the proteasome pathway. As one of the two main ways of protein degradation, ubiquitin proteolysis is essential for proteasomal degradation and plays an important role in the regulation of various biological processes. With the assistance of E1 ubiquitin-activating enzymes, E2 ubiquitin-conjugating enzymes and E3 ubiquitin ligases, the ubiquitin was activated and connected to the substrate protein, which was then ubiquitinated [35]. The E3 ubiquitin ligase TRIM39 as an PRDX3-associated protein prompted us to investigate whether TRIM39 mediated PRDX3 ubiquitination. We found that PRDX3 ubiquitination was markedly increased in renal fibrosis models and it was significantly weakened when TRIM39 was silenced. There are seven lysine (K) residues on the ubiquitin, and the fate of the substrate protein depends on its linked K residues. The effect of ubiquitination includes proteolytic and non-proteolytic related roles [36]. The ubiquitin chain attached to the lysine 63 (K63) residue is involved in DNA repair and signal transduction, while the K48 chain guides the substrate protein to the proteasome for degradation [37]. After the substrate protein binds to ubiquitin via K48, it is transferred to the proteasome for degradation. To investigate the ubiquitination mode of PRDX3, we constructed K6R, K11R, K27R, K33R, K48R and K63R mutants of ubiquitin for Co-IP assay, and our results showed that TRIM39 induced PRDX3 ubiquitination degradation through the K48-linked chain. We further constructed K52R, K73R, K83R, K149R, K217R, K241R and K248R mutants of PRDX3 to verify which residues were involved in PRDX3 ubiquitination degradation induced by TRIM39. And the Co-IP assay revealed that TRIM39 catalyzed the K48-linked polyubiquitination degradation of PRDX3-WT, PRDX3-K52R, PRDX3-K83R, PRDX3-K217R, PRDX3-K241R and PRDX3-K248R, but not that of PRDX3-K73R and PRDX3-K149R, which confirmed a vital role of TRIM39 in the ubiquitination degradation of PRDX3 at K73 and K149 for the first time.

In summary, we first found that TRIM39 induced the ubiquitination degradation of PRDX3 through the K48 chain, which resulted in the ROS accumulation, increased inflammatory cytokine production and renal fibrosis. And these effects were closely related to K73 and K149 of PRDX3. These findings provided vital and novel strategies for the therapy of renal fibrosis. Future development of TRIM39 inhibitors or PRDX3 agonists will be a crucial direction for renal fibrosis therapy.

## Materials and methods

### Animal experiments

This study was approved by the Research Ethics Committee of Renmin Hospital of Wuhan University (NO.WDRM20191006), and was performed according to guidelines for the Care and Use of Laboratory Animals.

Ten-week-old male C57BL/6 mice were provided by the center of experimental animals in the Medical College of Wuhan University (Wuhan, China). Animals were housed in a controlled environment with a 12-hour light-dark cycle and free access to food and water at temperature of 25°C, and randomly divided into two groups (*n* = 6 per group): control and UUO groups. The sample size of this study was calculated using ANOVA test. The results showed that 6 per group were required in this study to meet the significant differences in the groups using 80% performance test (1-β = 0.80), α = 0.05 error (95% confidence interval) with a two-sided hypothesis.

For the UUO model, C57BL/6 mice were fully anesthetized with sodium pentobarbital (60 mg/kg body weight), and a median incision was made. The left ureter proximal to the renal pelvis was ligated with 6-0 silk thread. Sham surgeries were performed in a similar way, except the ureter was not ligated. The animals were sacrificed 1, 4 or 7 days after UUO model establishment, and Sham and UUO kidneys were collected and stored at -80°C for subsequent biochemical studies and western blot analysis. TRIM39^Flox/Flox^ and Cre Cdh16 transgenic mice were purchased from Cyagen (Guangzhou, China). Kidney TRIM39 conditional knockout (CKO) mice were obtained by breeding TRIM39^Flox/Flox^ mice with Cre Cdh16 transgenic mice.

The following criteria are established in advance, if the knockdown or over-expression efficiency is not satisfied after verification, any experimental data from the mouse will not be used.

### Human kidney samples

All the human kidney samples were collected from patients at the Renmin Hospital of Wuhan University. All the patients signed informed consent and the process was approved by the Ethics Committee of Renmin Hospital of Wuhan University (Approval No. WDRM2022K-K075).

### Cell cultures

Human kidney-2 (HK-2) cells were purchased from China Center for Type Culture Collection (CCTCC, Wuhan, China). HK-2 cells were cultured under an atmosphere of 5% CO_2_ at 37 °C in DMEM medium (Invitrogen, USA) supplemented with 10% heat-inactivated fetal bovine serum. For renal fibrosis model, HK-2 cells were treated with 0, 1, 3, 5 ng/mL of TGF-β1. Human HEK293T cells were purchased from KeyGene BioTech (China) and cultured under an atmosphere of 5% CO_2_ at 37 °C in complete medium: DMEM (Invitrogen, USA) supplemented with 10% heat-inactivated FBS, and 1% penicillin-streptomycin (Beyotime, China).

### Real-time PCR analysis

Total RNA was isolated from kidney tissue and HK-2 cells using Trizol Reagent (ThermoFisher Scientific, Shanghai, China) according to the instructions of the manufacture, and revers transcribed into cDNA using a SuperScript First-strand Synthesis System (Invitrogen). The real-time PCR reactions (RT-PCR) were performed with GAPDH as internal control. The expression of mRNAs was shown as fold change relative to control and evaluated according to 2^-∆∆Ct^ method. PCR was performed using the primers in Supplementary Table 1.

### Western blot analysis

Renal tissues and HK-2 cells were lysed with RIPA buffer (Beyotime, Jiangsu, China) containing protease inhibitors to obtain total proteins. The cells were then centrifuged at 12000 rpm for 20 min at 4°C to remove cell debris. Protein was quantified using the BCA kit (abcam, shanghai, China). The lysed protein sample were separated on SDS polyacrylamide gel and then transferred to PVDF membrane. Subsequently, it was blocked with 5% skimmed milk, followed by overnight incubation at 4°C with the primary antibodies as follows: anti-PRDX3 (ab73349, abcam), anti-TRIM39 (ab227542, abcam), anti-α-SMA (ab7817, abcam), anti-collagen I (ab138492, abcam), anti-collagen IV (ab6586, abcam), anti-TNF-α (ab183218, abcam), anti-IL-6 (ab259341, abcam), anti-IL-1β (ab254360), anti-p-smad2(2224 R, YaJi Bio,shanghai, China), anti-smad2(K5306, YaJi), anti-p-smad3(3425, YaJi), anti-smad3(3484, YaJi), anti-p-samd4(AF8316, Affinity Bio), anti-samd4(AF0369, Affinity Bio), anti-p-smad7(AF3827), anti-smad7(AF5147) and anti-GAPDH (AB-P-R001, Hangzhou Goodhere Biotechnology Co., Ltd.). Then, the membranes were washed and incubated with the secondary antibody for 2 hours. Protein levels were quantified using the Image J software.

### Histology staining

The renal tissue sections were fixed with 10% formalin and embedded with paraffin, followed by hematoxylin and eosin (H&E) staining, Masson staining, and Sirius Red staining. The degree of fibrosis was assessed and quantified in five randomly selected microscopic fields. For H&E staining, paraffin sections were dewaxed and stained with hematoxylin, eosin, then dehydrated and visualized using a microscope. For Masson staining, Weigert ferriceuroxylin was stained for 10 min, followed by Masson blue and Ponceau S. Then the slices were washed and dehydration. For Sirius Red staining, sections were dewaxed and stained with Weigert ferriceuroxylin for 15 min, Then, it was washed and stained with Sirius Red for 1 h for visualization.

### DHE staining

10 μm unfixed frozen sections were treated with Dihydroethidium (DHE, 2μmol/L, Sigma) and incubated at 37 °C for 40 min without light. Then, the sections were washed with PBS for 3 times and detected by fluorescence microscope (Olympus IX51).

### Measurement of ROS

To measure the production of cytoplasmic ROS (cyto-ROS), HK-2 cells were rinsed with phosphate buffer saline (PBS), and stained with 10 μ M 2’,7’-Dichlorodihydrofluorescein diacetate (DCFH-DA, Beyotime Biotechnology, S0033S, China) at 37 °C for 30 min. For mitochondrial ROS (mito-ROS), Mitochondrial Reactive oxygen species (ROS) production rate test kit (YS2105A, YaJi) were used. Then, the cells were washed with PBS twice and observed by confocal laser microscope. For quantification, the fluorescent signals were converted into average grayscale intensities, which were subsequently analyzed using Image J software.

### Immunohistochemistry and immunofluorescence staining

4 μm slices were fixed with 4% paraformaldehyde for 20 min and blocked with 5% BSA for 1 hour. Next, they were incubated with anti-TRIM39 (ab227542, abcam) at 4 °C overnight. Then the sections were incubated with the secondary antibodies.

For immunofluorescence staining, HK-2 cells were fixed in 4% paraformaldehyde for 15 min after washing twice in PBS, and then blocked with 5% BSA for 30 min. The cells were incubated with primary antibodies of rabbit anti-PRDX3 (ab73349, abcam) and anti-TRIM39 (ab227542, abcam) overnight at 4 °C. The next day, fluorescent-conjugated secondary antibody (Boster Biological Technology, Wuhan, China) was used and incubated for 1 hour at room temperature in the dark. Cell nuclei were labeled by DAPI after washing twice with PBS. Finally, the sections were photographed by fluorescent confocal microscopy (Nikon, Tokyo, Japan).

### Small Interfering RNA (siRNA) Transfection

For PRDX3, Smad2, Smad3, Smad4 and Smad7 knockdown, small interfering RNA(SiRNA) of PRDX3 (Gene ID: 10935), Smad2 (Gene ID: 4087), Smad3(Gene ID:4088), Smad4 (Gene ID: 4089), Smad7 (Gene D: 4092) were designed. HK-2 cells were transfected with siRNA for 48 h through lipofectamine 3000 (Invitrogen, Carlsbad, CA, USA) before treated with TGF-β1. And the non-targeting siRNAs as a negative control (NC). Then, western blot was used to assess the effect of siRNA transfection.

### Adenovirus Transfection

For PRDX3 over-expression in vitro, HK-2 cells were transfected with adenovirus carrying human PRDX3 (Genepharma, Shanghai, China) at an MOI of 50 in DMEM for 10 hours, and then the medium was incubated in DMEM/F12 with 10% serum for 72 h, 48 hours after transfection, HK-2 cells were exposed to TGF-β1 or Control.

### Lentivirus Transfection

Stable knockdown cell lines were established by lentivirus carrying short hairpin RNA (shRNA) 48 h before exposure to TGF-β1 or no exposure (Control). The negative control was non-targeted shRNA (Sigma-Aldrich). The effect of TRIM39 and PRDX3 knockdown was verified by Western blot.

### Adeno-associated virus (AAV9) injection

For over-expression of PRDX3, the AAV9-packed PRDX3 over-expression plasm was injected to mice from the tail vein 2 weeks before surgery, and the control group was injected with AAV9 at the same time. The PRDX3 silencing in CKO mice was performed by injecting AAV9 carrying shRNA targeting PRDX3 from the tail vein 2 weeks before surgery, and the negative control group was injected AAV9 carried shRNA at the same time. Western blot was used to evaluate the efficiency of PRDX3 over-expression or silencing.

### Immunoprecipitation

HK-2 cells were removed from the incubator and rinsed twice with pre-cooled PBS. After treatment with lysis buffer solution, the cells were scratched and lysed. The lysed HK-2 cells were centrifuged at 1,4000 rpm and at 4 °C for 15 min, and collected the total protein. IgG and corresponding immunoprecipitation antibody were added, and then protein A/G was added and incubated at 4 °C for 3 hours. After immunoprecipitation, the precipitates were centrifuged at 4 °C for 3 min. The supernatant was sucked out, and the protein A/G was washed 4 times with 1 ml lysis buffer. Finally, the loading buffer was added to the precipitates and then boiled for 5 min. SDS-PAGE separation and Coomassie Brilliant Blue staining were performed following by western blot analysis.

### Co-immunoprecipitation

According to the above method, cells were collected in RIPA buffer, and 2 mg of cell proteins was pre-cleared. Cell lysates were incubated with anti-PRDX3 (ab73349, abcam) and anti-TRIM39 (ab227542, abcam) or control IgG at 4°C for 10 hours. Western blot analysis was performed after precipitation washing.

### Ubiquitination assay

To explore the ubiquitination chain of PRDX3, Myc-ubiquitin, HA-TRIM39, Myc-ubiquitin (K6R, P0584, miaolingbio, China), Myc-ubiquitin (K11R, P0689, miaolingbio), Myc-ubiquitin (K27R, P0585, miaolingbio), Myc-ubiquitin (K33R, P0587, miaolingbio), Myc-ubiquitin (K48R, P0588, miaolingbio), Myc-ubiquitin (K63R, P0854, miaolingbio) and Flag-PRDX3 expressing plasmids were used to transfect HEK293 cells for Co-IP assay; To explore the lysine residues associated with TRIM39-mediated ubiquitination of PRDX3 protein. Myc-ubiquitin, HA-TRIM39, Flag-PRDX3 (WT) and its mutants Flag-PRDX3 (K52R), Flag-PRDX3 (K73R), Flag-PRDX3 (K83R), Flag-PRDX3 (K149R), Flag-PRDX3 (K217R), Flag-PRDX3 (K241R) and Flag-PRDX3 (K248R) expressing plasmids were used to transfect HEK293 cells for Co-IP assay. The PRDX3 point mutation primers were listed in Supplementary Table 2.

### Statistical analysis

All the data was expressed as the means ± standard error of the mean, and the data was analyzed using Graphpad Prism 5.0 software. The differences between two groups were statistically analyzed using the Student’s t-test. Comparisons between multiple groups were analyzed using one-way analysis of variance. The *P* value < 0.05 was considered statistically significant.

### Supplementary information

SUPPLEMENTAL MATERIALS

## Data Availability

The data that support the findings of this study are available from the corresponding author (drwanglei@whu.edu.cn) upon reasonable request.
