# Supplementary material for: The E3 ubiquitin ligase TRIM39 modulates renal fibrosis induced by unilateral ureteral obstruction through regulating proteasomal degradation of PRDX3
Source: Cell Death Discov. 2024 Jan 9;10:17. doi: 10.1038/s41420-023-01785-4 (PMC10776755; doi:10.1038/s41420-023-01785-4)
Supplement: Supplementary file 1 — SUPPLEMENTAL MATERIALS [file 41420_2023_1785_MOESM1_ESM.docx]

**SUPPLEMENTAL MATERIALS**

**Supplementary Table 1. Prime sequence of genes**

| **Gene name** | **Sequence 5’-3’** | **Accession number** |
| --- | --- | --- |
| mouse *GAPDH* forward | TCTCCTGCGACTTCAACA | NM_001289726.2 |
| mouse *GAPDH* reverse | TGTAGCCGTATTCATTGTCA |  |
| human *GAPDH* forward | TATGACAACAGCCTCAAGAT | NM_001256799.3 |
| human *GAPDH* reverse | AGTCCTTCCACGATACCA |  |
| mouse *tnf-α* forward | CCCTCACACTCAGATCATCTTCT | NM_001278601.1 |
| mouse *tnf-α r*everse | GCTACGACGTGGGCTACAG |  |
| human *tnf-α* forward | GCCGCATCGCCGTCTCCTAC | NM_000594.4 |
| human *tnf-α* reverse | CCTCAGCCCCCTCTGGGGTC |  |
| mouse *il-6* forward | AGTTGCCTTCTTGGGACTGA | NM_001314054.1 |
| mouse *il-6* reverse | TCCACGATTTCCCAGAGAAC |  |
| human *il-6* forward | AGTTGCCTTCTTGGGACTGA | NM_000600.5 |
| human *il-6* reverse | TCCACGATTTCCCAGAGAAC |  |
| mouse *il1β* forward | ACCTTCCAGGATGAGGACATGA | NM_008361.4 |
| mouse *il1β* reverse | AACGTCACACACCAGCAGGTTA |  |
| human *il-1β* forward | TCAATGGCAATGAGGATG | NM_000576.3 |
| human *il-1β* reverse | TGTAGTGGTGGTCGGAGA |  |
| mouse *collagen I* forward | GTGGCGGTTATGACTTCA | NM_007742.4 |
| mouse *collagen I* reverse | CTGCGGATGTTCTCAATCT |  |
| human *collagen I* forward | GAGGGCCAAGACGAAGACATC | NM_000088.4 |
| human *collagen I* reverse | CAGATCACGTCATCGCACAAC |  |
| mouse *α-sma* forward | TGAAGAGCATCCGACACT | NM_007392.3 |
| mouse *α-sma* reverse | GCCTGAATAGCCACATACA |  |
| human *α-sma* forward | GGGGTGATGGTGGGAATG | NM_001141945.3 |
| human *α-sma* reverse | GCAGGGTGGGATGCTCTT |  |
| mouse *collagen IV* forward | GCTTGGTGCTTACTCTTAAC | NM_009931.2 |
| mouse *collagen IV* reverse | TGACAGACGGTTGGAATG |  |
| human *collagen IV* forward | ATAATCACACTGCGGTAGG | NM_000091.5 |
| human *collagen IV* reverse | TAAGGCGGTCAACAACTC |  |

**Supplementary Table 2. PRDX3 point mutation primers (Accession number: human *PRDX3*: NM_001302272.2)**

| **name** | **Sequence 5’-3’** |
| --- | --- |
| K52R forward | gaactggtgctgaataatcttgcttgactggaaccag |
| K52R reverse | ctggttccagtcaagcaagattattcagcaccagttc |
| K73R forward | cggctgtacccctaaaatagggtgcatgctggg |
| K73R reverse | cccagcatgcaccctattttaggggtacagccg |
| K83R forward | aggcttaggtctctgaactctccattgacaacggc |
| K83R reverse | gccgttgtcaatggagagttcagagacctaagcct |
| K149R forward | tggcccaaaccaccattccttcttggtgtatttatcc |
| K149R reverse | ggataaatacaccaagaaggaatggtggtttgggcca |
| K217R forward | ctggaacgccctcaccaagcggagggttt |
| K217R reverse | aaaccctccgcttggtgagggcgttccag |
| K241R forward | ctggacttggcctgatcgtaggagaatccggtg |
| K241R reverse | caccggattctcctacgatcaggccaagtccag |
| K248R forward | cttctgaaagtactctctggaagcagctggacttg |
| K248R reverse | caagtccagctgcttccagagagtactttcagaag |

**Supplemental Figure legends**


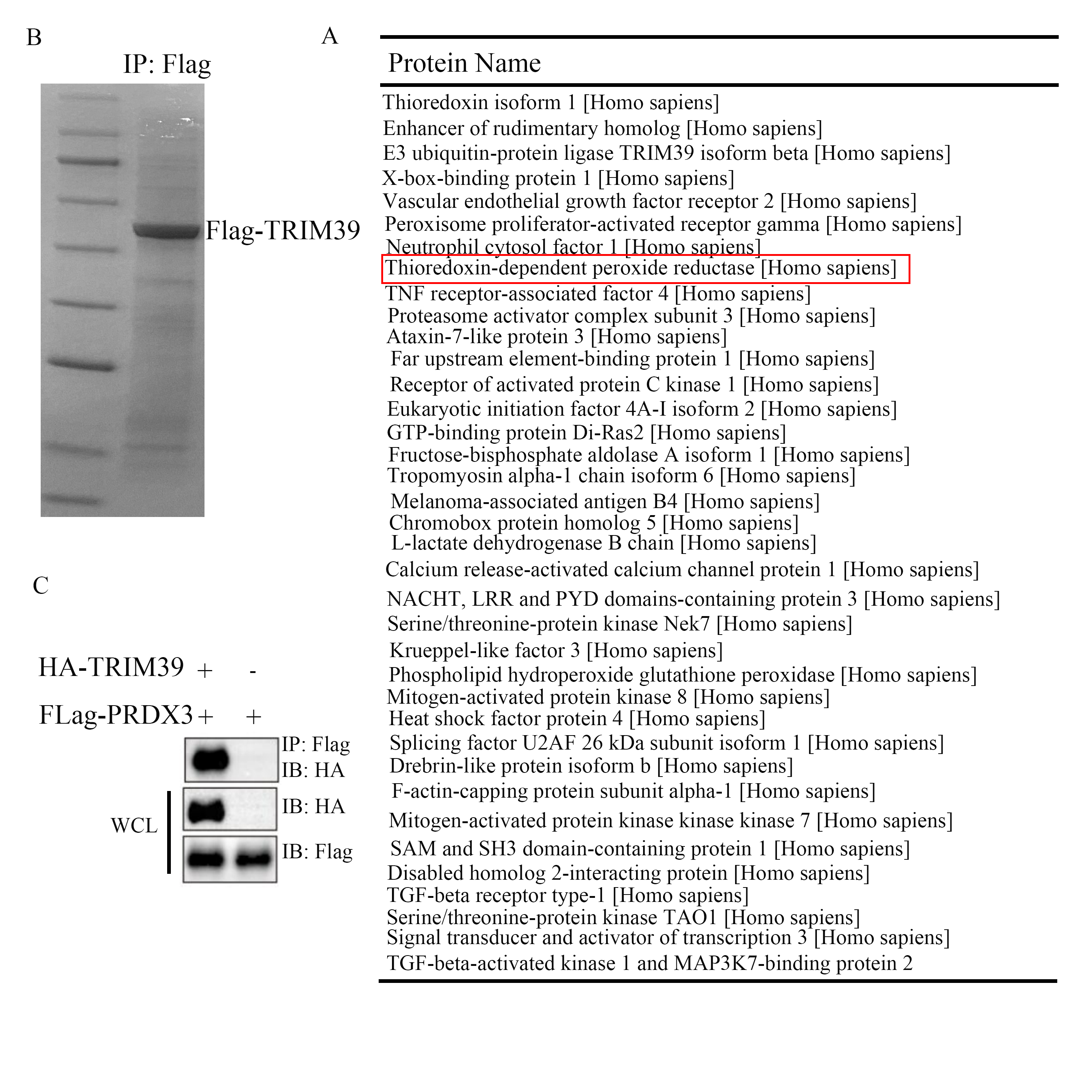


**Supplementary Figure S1. TRIM39 interacted with PRDX3**

(A).Mass spectrometry analysis of proteins interacted with TRIM39.

(B).Flag-TRIM39 pull-down products from the affinity purification using HEK293 cells were separated by SDS-polyacrylamide gel electrophoresis (SDS-PAGE) and visualized by Coomassie brilliant blue staining.

(C).Co-IP analysis in HK-2 cells transfected with plasmids expressing Flag-PRDX3, HA-TRIM39 to investigated whether TRIM39 interacted with PRDX3.

Each experiment was repeated independently for a minimum three times.


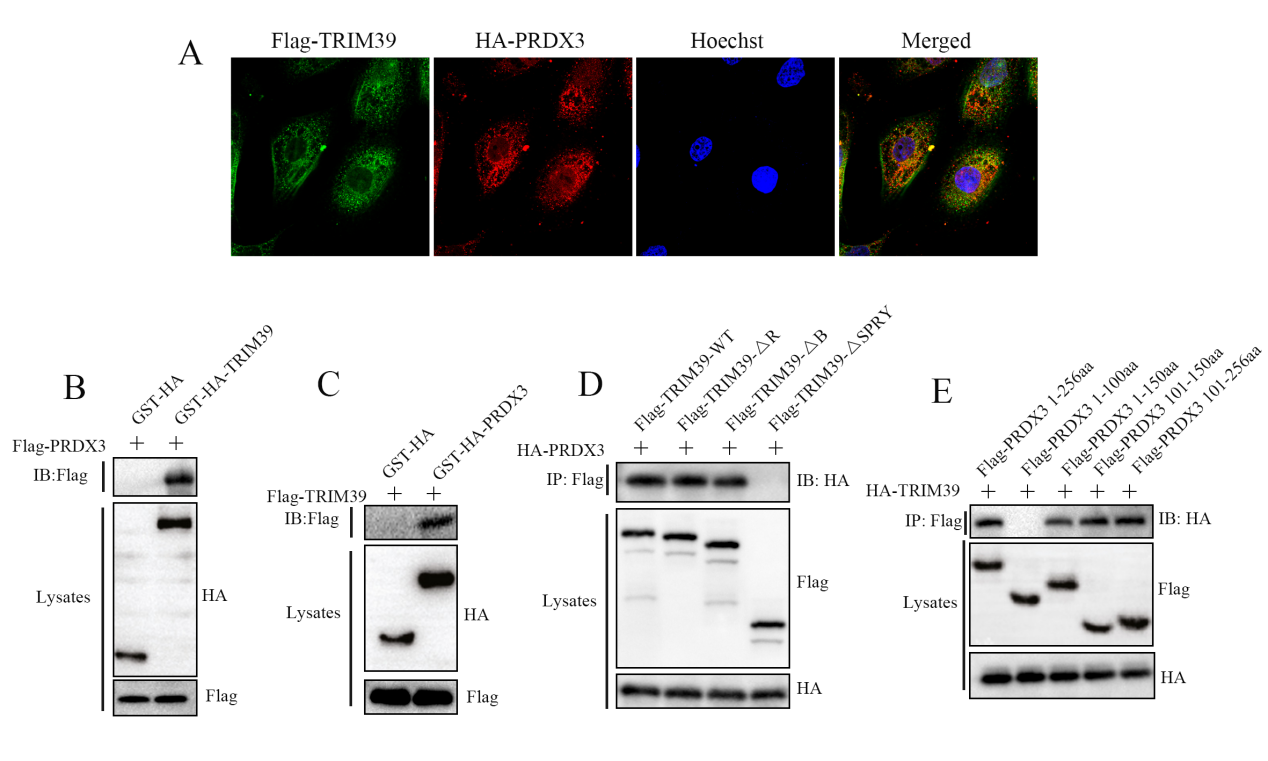


**Supplementary Figure S2.**  **TRIM39 colocalized with PRDX3, and the SPRY domain of TRIM39 interacted with the 101-256aa region of PRDX3 directly**

(A).Representative immunofluorescence staining images of colocalization of TRIM39 and PRDX3 in HK-2 cells.

(B,C). Glutathione S-transferase (GST) pull-down assay of the direct interaction between TRIM39 and PRDX3 translated by E.coli translation system in HK-2 cells.

(D).Co-IP analysis of the interaction of HA-PRDX3 with Flag-TRIM39 (WT) and TRIM39 truncation mutants (Flag-TRIM39-ΔR,Flag-TRIM39-ΔB, Flag-TRIM39-ΔSPRY) in HEK293 cells.

(E).Co-IP analysis of the interaction of HA-TRIM39 with Flag-PRDX3 (1-256aa) and PRDX3 truncation mutants (Flag-PRDX3 1-100aa,Flag-PRDX3 1-150aa, Flag-PRDX3 101-150aa, Flag-PRDX3 101-256aa) in HEK293 cells.

Each experiment was repeated at least three times independently.

**
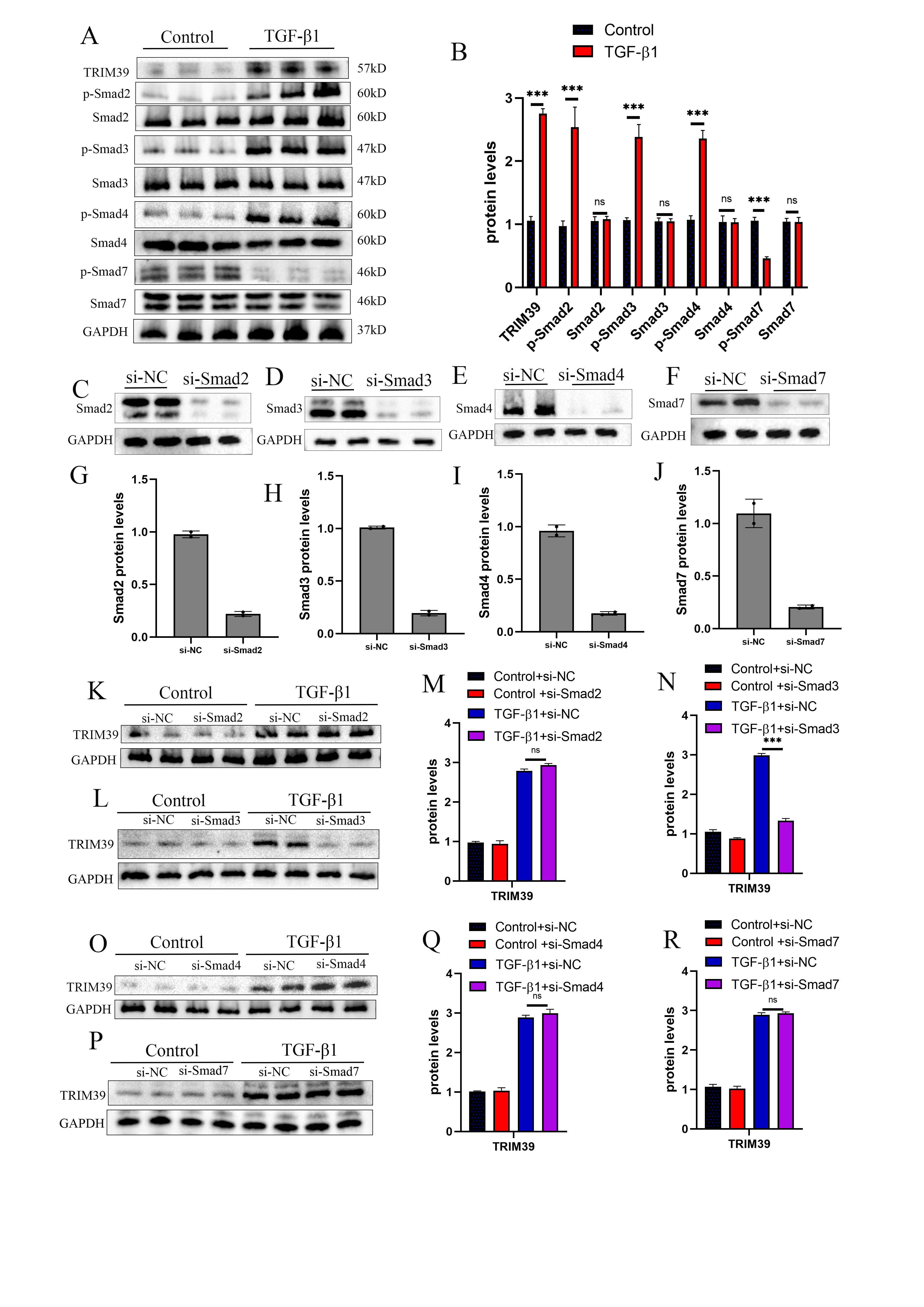
**

**Supplementary Figure S3. TGF-β1 promoted TRIM39 expression by activating SAMD3 phosphorylation.**

(A,B). Representative western blot and quantification images of TRIM39, p-Smad2, Smad2, p-Smad3, Smad3, p=Smad4, Smad4, p-Smad7 and Smad7 expression in HK-2 cells of two groups.

(C,G).Representative western blot and quantification images of Smad2 in si-NC and si-Smad2 groups.

(D, H). Representative western blot and quantification images of Smad3 in si-NC and si-Smad3 groups.

(E, I). Representative western blot and quantification images of Smad4 in si-NC and si-Smad4 groups.

(F, J). Representative western blot and quantification images of Smad7 in si-NC and si-Smad7 groups.

(K, M).Representative western blot and quantification images of TRIM39 in each group.

(L,N).Representative western blot and quantification images of TRIM39 in each group.

(O, Q).Representative western blot and quantification images of TRIM39 in each group.

(P, R).Representative western blot and quantification images of TRIM39 in each group.

All data were showed as mean ± SEM, Two-way analysis of variance was used. * P < 0.05, ** P < 0.01, *** P < 0.001. Each experiment was repeated at least three times independently.


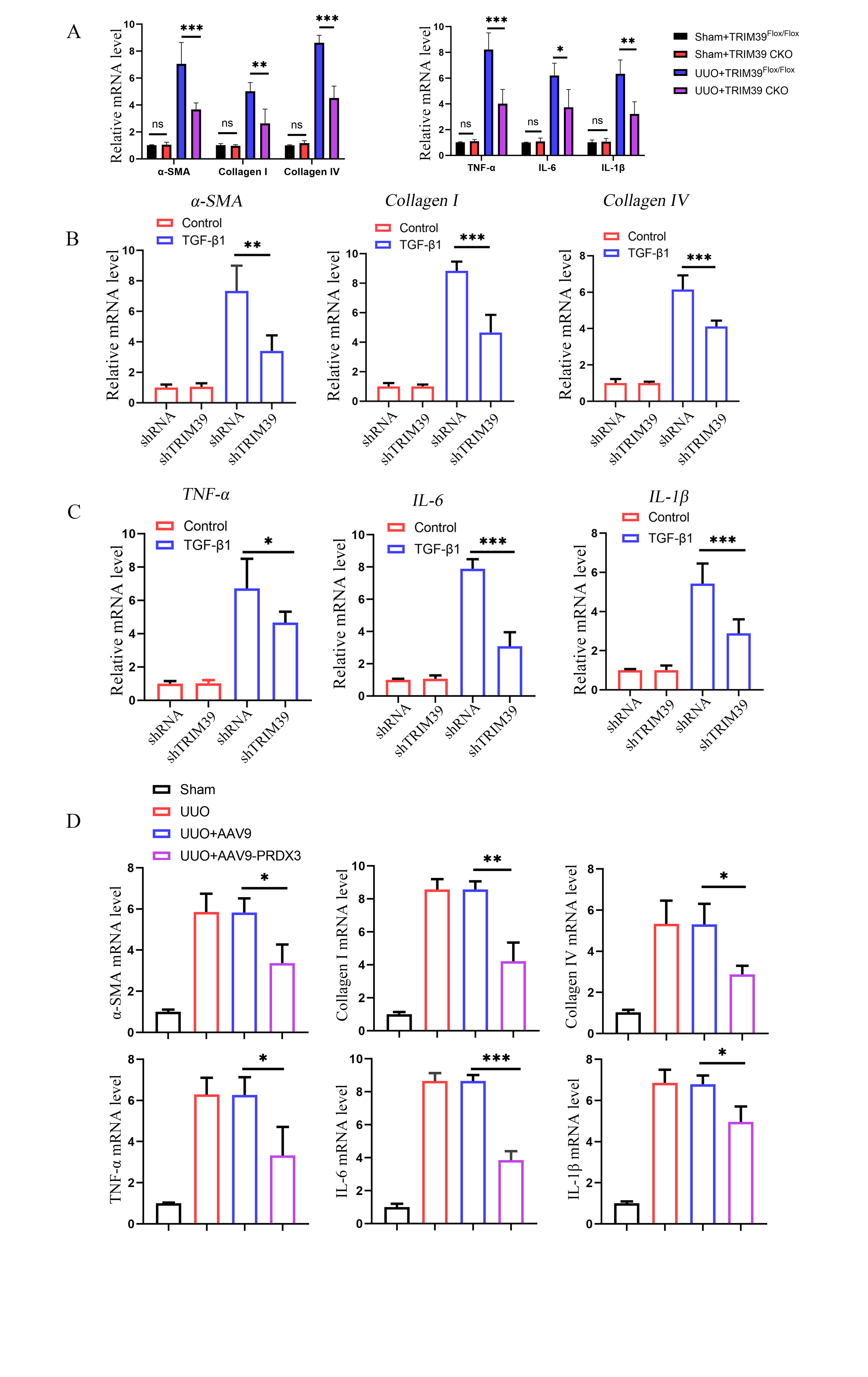


**Supplementary Figure S4.**  **TRIM39 knockdown or PRDX3 over-expression alleviated renal fibrosis.**

(A).Representative Real-time PCR analysis images for the relative mRNA levels of α-SMA, collagen I and IV, TNF-α, IL-6 and IL-1β in kidneys of the four groups (n=6).

(B, C).Real-time PCR analysis for the relative mRNA levels of α-SMA, collagen I and IV, TNF-α, IL-6 and IL-1β in HK-2 cells of each groups .

1. .Real-time PCR analysis for the expression of α-SMA, collagen I and IV, TNF-α, IL-6 and IL-1β in kidneys of the four mouse groups (n=6).

All data were showed as mean ± SEM, Two-way analysis of variance was used. * P < 0.05, ** P < 0.01, *** P < 0.001. Each experiment was repeated at least three times independently.
